# Association of the Model for End-Stage Liver Disease (MELD) Score With Spontaneous Bacterial Peritonitis in Patients With Liver Cirrhosis

**DOI:** 10.7759/cureus.112217

**Published:** 2026-07-07

**Authors:** Rafi Ullah, Jawad Ahmad, Saif Ur Rehman, Khalid Bashir, Saliha Nisar, Imran Khan

**Affiliations:** 1 Internal Medicine, West Cumberland Hospital, Whitehaven, GBR; 2 Internal Medicine, Lady Reading Hospital, Peshawar, PAK; 3 Internal Medicine, Khyber Teaching Hospital, Peshawar, PAK; 4 Acute and General Medicine, Queen Elizabeth Hospital Birmingham, Birmingham, GBR; 5 Internal Medicine, Pakistan Atomic Energy Commission, Islamabad, PAK

**Keywords:** end-stage liver disease, gastrointestinal bleed, liver cirrhosis, meld score, spontaneous bacterial peritonitis

## Abstract

Background: Spontaneous bacterial peritonitis (SBP) is a common and serious complication of decompensated liver cirrhosis, associated with increased morbidity, mortality, and prolonged hospitalization. Early identification of patients at high risk of SBP is essential to facilitate prompt diagnosis and timely treatment. The Model for End-Stage Liver Disease (MELD) score is a validated prognostic tool that reflects the severity of liver dysfunction and predicts mortality in patients with cirrhosis. However, its role in identifying patients at increased risk of SBP remains uncertain, particularly in resource-limited settings. This study aimed to determine the association between the MELD score and SBP in patients with liver cirrhosis.

Materials and methods: A case-control study was conducted in the Department of Medicine at Khyber Teaching Hospital, Peshawar, from March 15 to September 14, 2024. A total of 104 patients with liver cirrhosis and ascites (52 cases with SBP and 52 controls without SBP) were enrolled. SBP was defined as an ascitic fluid neutrophil count >250 cells/mm³. A MELD score >15 was defined as high. Data were analyzed using SPSS Statistics version 25 (IBM Corp. Released 2017. IBM SPSS Statistics for Windows, Version 25.0. Armonk, NY: IBM Corp.). Results: The mean age of the cases was 58.23 ± 8.51 years, and that of the controls was 53.27 ± 10.81 years. Hepatitis C virus was the leading etiology, accounting for 48.1% of cases. A high MELD score was observed in 36 (69.2%) cases and 18 (34.6%) controls. The association between a high MELD score and SBP was statistically significant (ꭓ² = 12.48, p < 0.001), with an odds ratio of 4.25 (95% CI: 1.90-9.53).

Conclusions: A high MELD score is significantly associated with the development of SBP in patients with liver cirrhosis. It serves as a reliable bedside clinical marker for identifying patients at higher risk of this severe complication.

## Introduction

Chronic liver disease (CLD) and cirrhosis are significant health problems worldwide [1,2]. There is a high prevalence of CLD in Pakistan, which has been linked to low socioeconomic status [3], poor sanitary infrastructure [4], and limited public awareness of liver disease risk factors [5]. In the region, more than 75% of liver cirrhosis and hepatocellular carcinoma are caused by viral hepatitis, namely, hepatitis B virus (HBV) and hepatitis C virus (HCV) [6]. Severe distortion of the liver architecture, portal hypertension, and subsequent hepatic decompensation occur due to progressive liver fibrosis.

Patients with decompensated cirrhosis and ascites are particularly vulnerable to developing spontaneous bacterial peritonitis (SBP) [7]. SBP is thought to result from "leaky gut" or from the passage of enteric bacteria through a damaged intestinal mucosal barrier [8]. This is exacerbated by impaired host immunity and decreased reticuloendothelial clearance in advanced liver disease [9]. SBP is a major turning point in the natural history of cirrhosis and is associated with significant risk of systemic sepsis and renal failure and in-hospital mortality rates of 10-20% [10-12].

In developing areas, where access to healthcare is often limited, it is crucial to identify patients most at risk of SBP so they can be treated promptly. The Model for End-Stage Liver Disease (MELD) score was developed to predict survival among patients undergoing transjugular intrahepatic portosystemic shunt procedures at the Mayo Clinic [13] and is now widely used as a prognostic indicator. The MELD score is based on objective laboratory values, avoiding the subjective clinical factors that comprise the traditional Child-Turcotte-Pugh (CTP) classification [14].

Increased baseline MELD scores are associated with the onset of SBP at a younger age and dramatically elevated mortality risk, as indicated by recent global literature [10]. However, current local information assessing this exact link in the Pakistani populace is very limited. This study aimed to assess the association between the MELD score and SBP in patients with liver cirrhosis, providing regional health authorities with evidence-based, up-to-date statistics to inform clinical management.

## Materials and methods

Study design and ethical considerations

This case-control study was conducted at the Department of Medicine, Khyber Teaching Hospital, Peshawar, over a six-month period from March 15 to September 14, 2024. The Institutional Research and Ethical Review Board of Khyber Medical College, Peshawar, approved the study (approval number: 919/DME/KMC), and informed consent was obtained from all participants; the study's purpose, risks, and benefits were explained to them.

Inclusion criteria required a diagnosis of liver cirrhosis for more than five years and the presence of hepatic ascites. This duration was selected to ensure inclusion of patients with established chronic hepatic decompensation. While this criterion potentially introduces selection bias by excluding patients with early-stage cirrhosis, it allowed for a more homogeneous cohort of end-stage liver disease patients.

Exclusion criteria included patients who received antibiotics in the preceding two weeks; used proton pump inhibitors (PPIs) due to their role as an independent risk factor for small intestinal bacterial overgrowth; had secondary bacterial peritonitis, hepatocellular carcinoma, extra-abdominal infections, immunosuppressive therapy, chronic kidney disease requiring dialysis, recent abdominal surgery, or incomplete clinical records.

Study participants

The sample size of 104 patients (52 cases and 52 controls) was calculated using OpenEpi software (Dean AG, Sullivan KM, Soe MM. OpenEpi: Open Source Epidemiologic Statistics for Public Health, Version. www.OpenEpi.com, updated 2013/04/06), based on an anticipated 35% proportion of low MELD scores (≤15) in controls and 10% in cases, with 80% power, an alpha of 0.05, and a 1:1 ratio. This ensured adequate power to detect a significant difference. A non-probability consecutive sampling method was used.

Cases comprised patients diagnosed with SBP, defined as an ascitic fluid polymorphonuclear leukocyte count >250 cells/mm³ in the absence of a surgically treatable intra-abdominal source of infection.

The control group comprised cirrhotic patients with ascites admitted during the same study period who underwent diagnostic paracentesis but had no evidence of SBP. Controls had an ascitic fluid PMN count of <250 cells/mm³, no positive clinical findings suggestive of SBP (including fever, abdominal pain or tenderness, altered mental status, or unexplained worsening of renal function attributable to SBP), and no microbiological or radiological evidence of intra-abdominal infection. Controls were selected from the same hospital population as the cases and represented patients with comparable underlying liver disease who differed only in the absence of SBP. To minimize selection bias, controls were enrolled consecutively during the study period and were comparable to cases with respect to age, sex, and the presence of cirrhosis with ascites.

Cirrhosis was diagnosed clinically (liver span <10 cm on percussion), biochemically (serum albumin <3 g/dl and prothrombin time >14 seconds), and radiologically (ultrasound showing a shrunken, nodular liver with coarse echotexture and an elevated caudate-to-right lobe ratio).

Clinical scores and definitions

SBP was diagnosed based on an ascitic fluid neutrophil count of ≥250 cells/mm³, which is the definitive diagnostic criterion regardless of clinical presentation. Diagnostic paracentesis was performed in all enrolled patients, and ascitic fluid analysis was used to determine the neutrophil count. While fever (≥38°C) and abdominal pain were recorded as clinical manifestations, they were not required for the definitive diagnosis, as SBP can present asymptomatically. Overall disease severity was evaluated using the CTP score [14]. Blood samples drawn at admission were used to measure bilirubin, creatinine, and INR. The MELD score was calculated using the standard version [13]:

\( 3.78 \times \ln(\text{bilirubin} \,[\mathrm{mg/dL}]) + 11.2 \times \ln(\mathrm{INR}) + 9.57 \times \ln(\text{creatinine} \,[\mathrm{mg/dL}]) + 6.43 \)

A threshold of >15 was chosen as the high MELD score, as this is the widely accepted clinical cutoff for prioritizing patients for liver transplantation evaluation.

Statistical analysis

Data were analyzed using SPSS Statistics version 25.0 (IBM Corp. Released 2017. IBM SPSS Statistics for Windows, Version 25.0. Armonk, NY: IBM Corp.). Frequencies and percentages were computed for categorical variables, while means and standard deviations were calculated for continuous variables. Differences in baseline characteristics were evaluated using the independent-samples t-test and the Chi-square test. The association between the MELD score and SBP was evaluated using the chi-square test, with odds ratios (OR) and 95% confidence intervals (CI) calculated. Subgroup analyses stratified by age, gender, and CTP class were performed using the chi-square test. A multivariable logistic regression model was also used to assess the association between a high MELD score and SBP, adjusting for age, gender, etiology, and CTP class. A p-value of ≤0.05 was considered statistically significant.

## Results

A total of 104 patients were enrolled, comprising 52 cases with SBP and 52 controls without SBP. The baseline demographic and clinical characteristics of the study population are summarized in Table 1. The mean age of the cases was 58.23 ± 8.51 years, significantly higher than that of the control group (53.27 ± 10.81 years; p = 0.010). Males constituted the majority in both groups, accounting for 36 (69.2%) of the cases and 32 (61.5%) of the controls.

**Table 1:** Baseline demographics and clinical characteristics of the study population (N = 104) * Indicates statistical significance at p < 0.05. SD: standard deviation, SBP: spontaneous bacterial peritonitis, CLD: chronic liver disease, HCV: hepatitis C virus, HBV: hepatitis B virus, CTP: Child-Turcotte-Pugh

Parameter	Cases (with SBP) n = 52	Controls (without SBP) n = 52	p-value
Age (years)			
Mean ± SD	58.23 ± 8.51	53.27 ± 10.81	0.010*
Gender, n (%)			
Male	36 (69.2%)	32 (61.5%)	0.536
Female	16 (30.8%)	20 (38.5%)	
Etiology of CLD, n (%)			
HCV	25 (48.1%)	17 (32.7%)	0.238
HBV	21 (40.4%)	25 (48.1%)	
Unknown	6 (11.5%)	10 (19.2%)	
CTP class, n (%)			
Class A	8 (15.4%)	11 (21.2%)	0.463
Class B	29 (55.8%)	31 (59.6%)	
Class C	15 (28.8%)	10 (19.2%)	

Regarding the etiology of liver cirrhosis, HCV was the most prevalent underlying cause, identified in 25 cases (48.1%) and 17 controls (32.7%). Evaluation of overall disease severity by the CTP score revealed that the majority of participants fell into Class B (55.8% of cases and 59.6% of controls). There were no statistically significant differences in gender, etiology, or CTP class distributions between the two groups.

The primary outcome analysis was performed by comparing the numbers of patients with MELD score >15 and MELD score ≤15 between the two groups. As shown in Table 2, in the case group, 36 (69.2%) patients had a high MELD score, whereas only 18 (34.6%) of the controls without SBP did. On the other hand, 16 (30.8%) cases and 34 (65.4%) controls had a low MELD score.

**Table 2:** Association of MELD score categories with SBP * Indicates statistical significance at p < 0.05. MELD: Model for End-Stage Liver Disease, SBP: spontaneous bacterial peritonitis, OR: odds ratio, CI: confidence interval

MELD score category	Cases (with SBP) n = 52	Controls (without SBP) n = 52	Test statistic (χ²)	p-value	OR (95% CI)
High MELD (>15)	36 (69.2%)	18 (34.6%)	12.48	<0.001*	4.25 (1.90-9.53)
Low MELD (≤15)	16 (30.8%)	34 (65.4%)			

A high MELD score was significantly associated with the presence of SBP (χ² = 12.48, p < 0.001) in the statistical analysis. Patients with a MELD score >15 had a significantly higher OR (4.25; 95% CI 1.90-9.53) of SBP compared with those with a MELD score ≤15. Stratified analysis showed that the association between a high MELD score and SBP remained significant across most subgroups: age ≤50 years (p = 0.002), age >50 years (p = 0.010), female sex (p < 0.001), and CTP class B (p = 0.010). The association did not reach significance among male patients (p = 0.158) or in CTP classes A (p = 0.125) and C (p = 0.096), likely reflecting reduced statistical power in these smaller strata rather than true effect modification. Additionally, after adjusting for age, gender, etiology, and CTP class in a multivariable logistic regression model, a high MELD score remained independently associated with SBP (adjusted OR 3.96; 95% CI 1.66-9.49; p = 0.002), and age was independently associated with SBP as well (adjusted OR 1.05; 95% CI 1.00-1.10; p = 0.045).

## Discussion

SBP is a serious complication of decompensated liver cirrhosis, which is strongly linked to morbidity and mortality [1,7]. In this study, the association between a MELD score and SBP was studied in the Pakistani population. Our findings showed that a high MELD score (>15) was strongly associated with SBP.

This association was confirmed by computing the OR of 4.25. This is in line with available literature from around the world [10,11]. Similarly, Gayatri et al. found that severe liver cirrhosis (MELD score >18) was significantly associated with SBP, further supporting the link between higher MELD scores and the development of SBP [15].

Several other international studies have similarly demonstrated the relationship between worsening liver disease severity and the occurrence of SBP. Tsung et al. concluded that a higher MELD score along with lower ascitic glucose levels are associated with higher mortality rates in cirrhotic patients with SBP [16]. Likewise, Almdal and Skinhoj reported that higher MELD scores were associated with poorer prognosis and increased short-term mortality among patients with SBP [17]. These studies underscore the utility of the MELD score not only as a prognostic scoring system for liver transplantation prioritization but also as a practical clinical tool for early identification of cirrhotic patients at high risk of infectious complications.

Our findings are particularly relevant in the South Asian setting, where viral hepatitis remains the predominant etiology of chronic liver disease. In contrast, many Western studies report alcohol-related cirrhosis as the leading underlying cause associated with SBP [9]. Despite these etiological differences, the association between higher MELD scores and SBP appears consistent across populations, suggesting that the severity of hepatic dysfunction itself is the principal determinant of infection risk rather than the underlying cause of cirrhosis alone [18].

Furthermore, some studies have proposed MELD score thresholds ranging from 15 to 20 for predicting SBP and adverse outcomes in cirrhotic patients [11,19]. The present study demonstrated a significant association with a MELD score >15, which is clinically important because it may help clinicians identify high-risk patients earlier and consider closer surveillance and prompt diagnostic paracentesis.

Compared with traditional prognostic models such as the CTP score, the MELD score may provide superior risk stratification because it objectively incorporates renal function via serum creatinine levels. Since renal impairment frequently accompanies SBP and significantly worsens prognosis, as in the case of hepatorenal syndrome, the MELD score may better reflect the overall physiological deterioration associated with advanced cirrhosis and systemic infection [20].

Several prognostic models have been developed to improve risk stratification in patients with decompensated cirrhosis beyond the conventional MELD score. The MELD-Na score incorporates serum sodium and has demonstrated superior prediction of short-term mortality by accounting for the adverse prognostic impact of hyponatremia. More recently, the MELD 3.0 score further improves predictive accuracy by incorporating serum albumin and sex into the original MELD score. Likewise, the Chronic Liver Failure Consortium Acute-on-Chronic Liver Failure (CLIF-C ACLF) score provides excellent prognostic discrimination in patients with acute-on-chronic liver failure by integrating organ failure assessment with age and white blood cell count. Although these scores were not evaluated in the present study, they may offer improved prognostic performance in selected populations. Future prospective studies should compare the MELD score with the MELD-Na, MELD 3.0, and CLIF-C ACLF scores to predict SBP and related clinical outcomes and to determine the most appropriate risk stratification tool for cirrhotic patients.

The pathogenesis of this clinical association is thought to be due to the gradual decline of liver function and the systemic immunologic effects of that decline [9]. Patients with advanced cirrhosis suffer from severe hepatic decompensation and altered gut microbiota integrity, which adversely affects the integrity of the intestinal mucosa. This increased permeability allows for enteric bacteria to be translocated into the peritoneal cavity [8]. Moreover, as the MELD score increases, renal and hepatic function decline, and these translocated bacteria are cleared by the reticuloendothelial system, which also deteriorates with increasing MELD scores [7]. This makes SBP a manifestation of advanced end-stage liver failure rather than merely a localized infection.

Of particular interest, viral hepatitis (HCV and HBV) was the most common cause in our population, which reflects the context in which we live in Pakistan [6,21]. Alcohol consumption in our population is low, and viral hepatitis is the leading cause of chronic liver disease; this is in line with other studies elsewhere in the country; however, some studies report a higher prevalence of SBP in the alcohol-related cirrhosis population [12].

The MELD score has significant clinical benefits over the CTP score for predicting these complications. Unlike the clinical grading of ascites and encephalopathy, all values used in the MELD score are objective and reproducible data. Most importantly, the use of serum creatinine in the MELD equation accounts for renal dysfunction, which is a major prognostic determinant in cirrhosis and is a common and life-threatening complication of SBP, hepatorenal syndrome.

This study has a few limitations that must be acknowledged. First, the case-control study design is observational and cross-sectional, which establishes associations but cannot confirm temporal causality or true predictive performance. Second, diagnostic paracentesis and MELD scores were evaluated simultaneously at admission, making it difficult to determine whether the high MELD score preceded the infection or was acutely elevated as a result of the SBP itself (reverse causation). Third, while we adjusted for age, gender, etiology, and CTP class in a multivariable model, we were unable to adjust for other potential confounders not captured in our dataset, including previous gastrointestinal bleeding, ascitic protein levels, and prior antibiotic exposure. Finally, patients taking PPIs were excluded, which strengthens our internal validity but may limit the generalizability of these findings to the broader cirrhotic population who frequently use these medications.

## Conclusions

The MELD score is a strong, objective predictor of SBP in patients with liver cirrhosis. Patients with a MELD score >15 have much higher odds of this serious infection. The quick calculation of the MELD score can help physicians define those at high risk at the bedside, monitor them more carefully, and respond to treatment more quickly to avoid fatal complications.
